# Osteopontin levels are associated with late-time lower regional brain volumes in multiple sclerosis

**DOI:** 10.1038/s41598-021-03173-3

**Published:** 2021-12-08

**Authors:** Gergely Orsi, Zsofia Hayden, Tamas Cseh, Timea Berki, Zsolt Illes

**Affiliations:** 1 MTA-PTE Clinical Neuroscience MR Research Group, Eötvös Loránd Research Network (ELKH), Ret str. 2, 7623 Pecs, Hungary; 2 Department of Neurology, Medical School, University of Pecs, Pecs, Hungary; 3 Department of Immunology and Biotechnology, Medical School, University of Pecs, Pecs, Hungary; 4 Department of Neurology, Odense University Hospital, Odense, Denmark; 5 Department of Clinical Research, University of Southern Denmark, Odense, Denmark

**Keywords:** Neurology, Neurological disorders

## Abstract

Osteopontin (OPN) is a proinflammatory marker produced by systemic immune and central nervous system (CNS) resident cells. We examined, if the level of OPN in the cerebrospinal fluid (CSF) and blood is associated with late-time regional brain volumes and white matter (WM) lesion load in MS. Concentrations of OPN in blood and CSF were related to MRI findings 10.1 ± 2.0 years later in 46 patients with MS. OPN concentration was measured by ELISA, while regional brain volumes and lesion load was assessed by magnetic resonance imaging (MRI) using 3D MPRAGE sequence and automated MR volumetry. OPN measured in the CSF was associated with several regional brain volumes and WM lesion load measured 10.1 ± 2.0 years later. CSF OPN concentration correlated with long-term enlargement of lateral- and inferior lateral ventricles and the elevation of gross CSF volume, in conjunction with the reduction of several cortical/subcortical gray matter and WM volumes. Serum OPN showed no long-term association with regional brain volumes. OPN measured from the CSF but not from the serum was associated with lower regional brain volumes measured a decade later, indicating the primary role of inflammation within the CNS in developing long-term brain related alterations.

## Introduction

Multiple sclerosis (MS) is an inflammatory, demyelinating, and neurodegenerative disease of the central nervous system. It is the most frequent non-traumatic cause of permanent neurological disability in young and middle-aged adults^1,2^. The main driver of the pathology is central nervous system (CNS) inflammation in both the white matter (WM) and gray matter (GM) that induces a number of pathological events ultimately leading to progressive disability in part of the patients^3,4^. Magnetic resonance imaging (MRI) is a distinguished paraclinical investigation in the process of clinical diagnosis of MS, along with cerebrospinal fluid- (CSF) and blood tests, and MRI is also basic in defining the clinical course of MS^5^.

MRI is a sensitive tool for detecting MS related tissue abnormalities in the CNS, especially the brain-related focal white matter (WM) and gray matter (GM) lesions, as well as the diffuse, or localized tissue loss (atrophy)^6,7^. Brain atrophy was shown to be extensive in MS, with nearly 0.5–1.35% brain volume loss/year, much higher than that of normal aging (0.1–0.5%/year)^8^. It arises early in the course of the disease and accelerates along with disease progression^9^.

Osteopontin (OPN), also known as early T cell-activation gene 1 or secreted phosphoprotein 1 (SPP1) was originally identified as a bone matrix protein. OPN was shown to act as a pro-inflammatory cytokine in several autoimmune diseases, most notably in neuromyelitis optica spectrum disease^10^ and MS^11^. OPN is produced by various immune cells, including T cells, B cells, macrophages, dendritic cells, and natural killer cells.

Proinflammatory Th17 immune responses induced by OPN have been indicated in the pathogenesis of MS^12^. Enhanced OPN expression was found in active MS lesions^13^, in microvascular endothelial cells and macrophages of plaques and also in the white matter surrounding the plaques^14^. OPN has a prominent role in secondary neurodegeneration: microglia secrete OPN into the extracellular matrix, which activates and recruits macrophages and CNS resident cells that modulate inflammatory responses^15,16^. A recent meta-analysis by Agah et al. summarizes the findings on OPN levels in multiple sclerosis; OPN level in the CSF is higher in MS compared to healthy controls^17^. Serum OPN levels were shown to be elevated in all MS subtypes, except for clinically isolated syndrome^17^. The higher concentration of OPN in CSF compared to serum suggests OPN expression by CNS cells^18^ . The elevated CSF and serum OPN levels were shown in both relapsing–remitting MS (RRSM) and secondary progressive MS (SPMS) patients^19^, moreover, higher OPN levels in CSF were measured in patients with active disease, as compared to patients with stable disease^17^. The highest OPN concentration in CSF was measured in RRSM patients^17^. OPN levels in the CSF correlate with development of microstructural abnormalities and functional connectivity within 10 years^20^. Therefore, we here examined the long-term effect of OPN on regional brain volumes in patients with MS.

## Results

**Figure 1:**
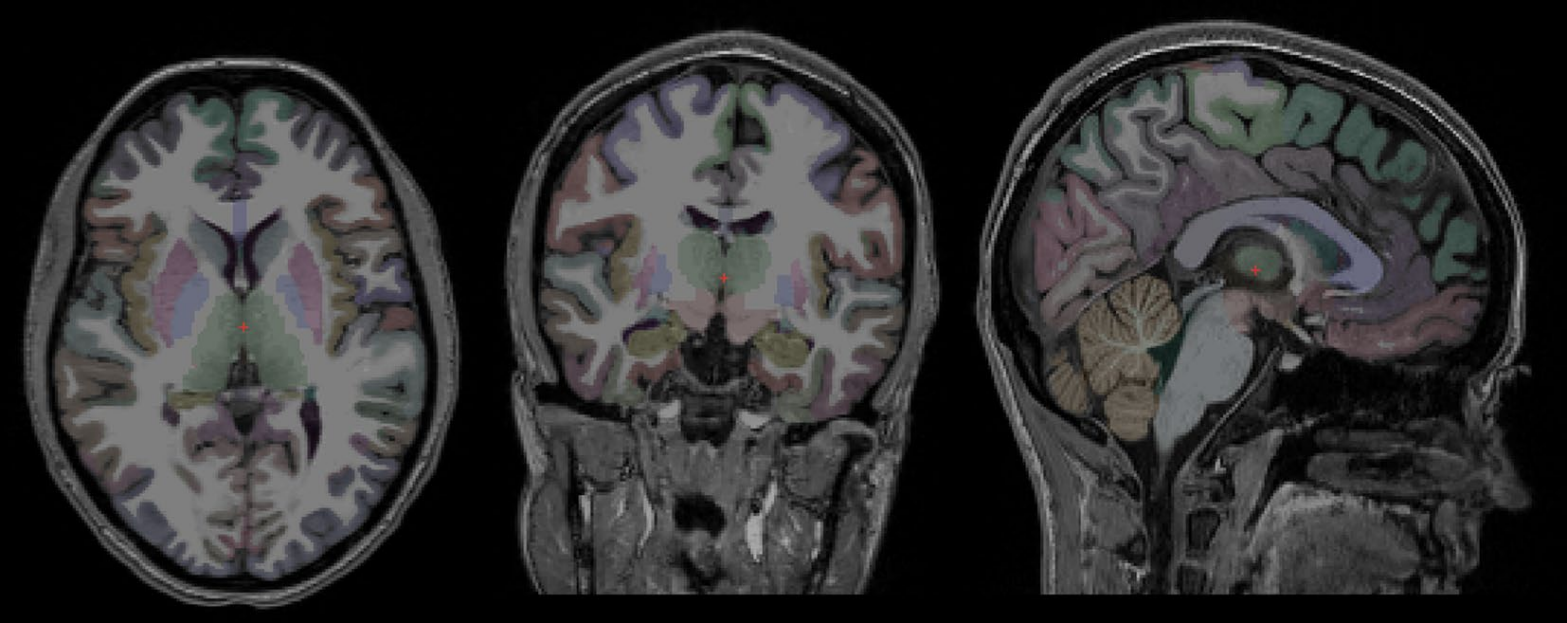
From left to right: axial, coronal, and sagittal representative images showing the results of automatic segmentation. All images are shown in radiologic convention.

40 reliably segmented structures were extracted from Freesurfer’s segmentation output, omitting brainstem. Figure 1 shows the results of automatic segmentation on a randomly selected subject. Multiple linear regression models included age, gender, and estimated total intracranial volume as variables of no interest. Dependent variables were the segmented brain structures and OPN was included as independent variable of interest in separate models. Storage time was also included in the initial models, but was removed from the final ones, as storage time was not proved to be a significant predictor in any of the tested models.

OPN measured from serum samples showed no associations with the segmented volumes, regardless of the time of collection [median OPN concentration 12.52(9.0–16.7) ng/mL at the time of CSF collection and 9.8(6.2–18.8) ng/mL at the time of MRI].

**Figure 2:**
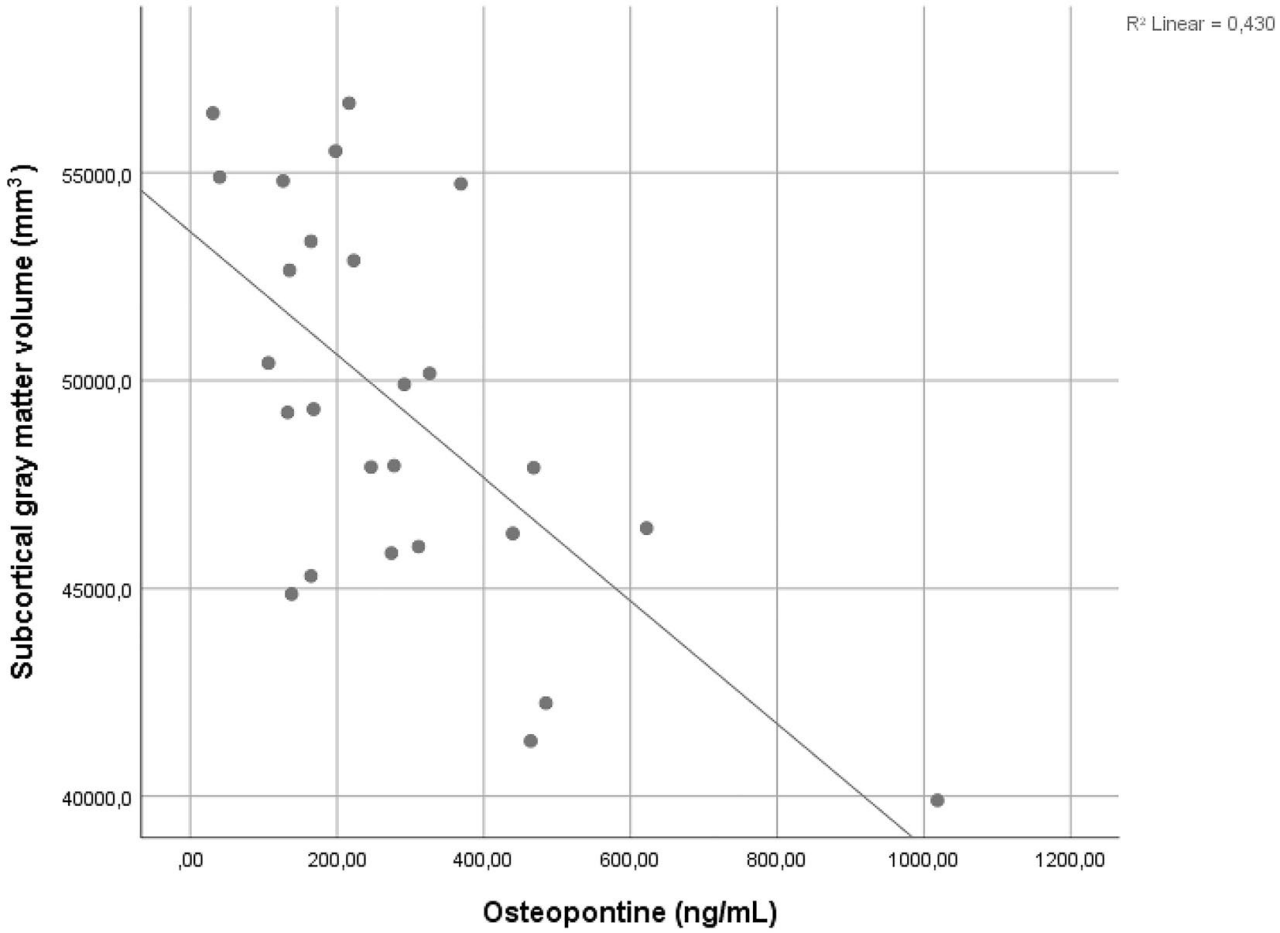
Correlation between osteopontin concentration measured from cerebrospinal fluid and subcortical gray matter volume measured 10.1 ± 2.0 years after lumbar punction. Pearson’s r = −0.579, p = 0.00196. Variables are unadjusted for age, gender, and estimated total intracranial volume and only serves demonstrational purposes. Associations between the measured variables, appropriately adjusted for the nuisance factors, are shown in Table 2.

OPN measured from CSF collected 10.1 ± 2.0 years before MRI [median 246.4(164.5–439.5) ng/mL] showed highly significant and diverse associations with the segmented brain volumes. CSF OPN levels were positively associated with the volumes of the ventricles and CSF; left- and right inferior lateral ventricles (t = 3.318, p = 0.0013 and t = 5.012, p = 0.00005, respectively), left- and right lateral ventricles (t = 3.272, p = 0.0036 and t = 3.345, p = 0.0031, respectively), T1 derived total WM lesion volume (t = 2.991, p = 0.007), and CSF volume (t = 3.055, p = 0.006). Besides, inverse associations were found between CSF OPN levels and the following regional brain volumes: subcortical GM volume (t = −4.03, p = 0.0006), left- and right ventral diencephalon (t = −3.69, p = 0.0014 and t = −3.425, p = 0.0025), ventricle-free supratentorial volume (t = −3.341, p = 0.0031), total cerebral cortex and WM volumes (t = −2.768, p = 0.0115 and t = −2.894, p = 0.0087, respectively). More details and further associations are shown in Table 2. Figure 2 shows the raw correlation between osteopontin concentration measured from cerebrospinal fluid and subcortical gray matter volume measured 10.1 ± 2.0 years later.

## Discussion

OPN measured from the CSF was associated with reduced brain volume in several regions within 10.1 ± 2.0 years, indicating that the level of CSF OPN was associated with regional brain volumesmeasured a decade later. Results showed that an elevated CSF OPN concentration predicted the late-time enlargement of lateral- and inferior lateral ventricles and the elevation of gross CSF volume, in conjunction with the reduction of several cortical and subcortical GM volumes. Tortorella et al. conducted a cross-sectional study on patients with clinically isolated syndrome (CIS), measuring OPN concentrations from CSF and gross brain volumetry, including total GM volume, peripheral GM volume, total brain volume, ventricular volume and manually assessed corpus callosal index. Their results showed that OPN levels were only weakly associated with corpus callosum index^21^. In our longitudinal cohort, CIS patients were not included, and we did not observe such associations in our MS populations. Moreover, cross-sectional statistical analyses yielded no significant associations at all, and all significant correlations surviving FDR correction corresponded to OPN concentration from CSF and MRI acquired 10.1 ± 2.0 years later. The suggestion that OPN may be associated with WM damage^21^ is well supported by our previous study in the same cohort, demonstrating that CSF OPN levels are related to wide-spread WM alterations localized to the normal appearing white matter (NAWM) of left superior and inferior longitudinal fasciculi, external capsule, forceps minor (genu of corpus callosum) and anterior corona radiata, indicating myelin loss and axonal degenerations^20^. These previous and the current data may thus suggest that patients with higher OPN CSF levels developed more extensive WM damage accompanied by the association with reduced WM and GM regional volumes. Indeed, GM atrophy is strongly associated with WM injury in MS patients, particularly with injury to association fibers^22^.

It is conceivable that soluble biomarkers, specific for late-time regional brain volumes, alone or in combination with MRI biomarkers, may be clinically valuable in prognostic evaluation at the beginning of MS disease. Brain atrophy has clinically relevant impact on MS pathogenesis: higher atrophy rate leads to the worsening of expanded disability status scale (EDSS) and progression to disability^23^. Recently, several phase III trials defined brain atrophy as an outcome in both relapsing and progressive MS, and a number of disease modifying treatments significantly reduced atrophy rate^24^. If confirmed in other independent cohorts, CSF OPN concentration may be a potential marker for screening patients for high risk of accelerated atrophy rate in the long-term. Despite the fact that due to study design, we cannot state that the observed association with smaller regional brain volumes (and larger ventricles) indicate atrophy, our study may still indicate that in the development of brain volume losses reported earlier^8,9^, OPN produced within the CNS plays an important role. This also emphasizes the role of inflammation within the CNS compartment in the evolution of atrophy.

## Methods

### Subjects

Forty-six patients with clinically definitive MS (32 females, age range at MRI: 20–68 years) have participated in the study. Serum and CSF samples were collected 10.1 ± 2.0 years before MRI and aliquots were kept at − 80 °C until further processing. A new serum sample was taken at the day of MRI acquisition. All patients participating in the study had MS fulfilling the 2017 modified McDonald diagnostic criteria^25^. In case of relapsing MS, the MRI measurements were taken in the remission phase. Most of the patients were on chronic disease modifying treatment (Table 1). 11% of the patients had primary progressive MS, and at the time of MRI, 67% had relapsing and 22% secondary progressive MS. During the follow-up period (10.1 ± 2.0 years), the median number of relapses was 3 (IQR:2–4), and EDSS has increased in the study population (p = 0.034, Wilcoxon Signed Rank Test).

**Table 1:** Clinical characteristics of MS patients.

Characteristics	Number of patients, mean ± SD or median (IQR)
**Demographics**	
Number of patients	46
Disease duration (years)	12.5 (8.75–15.25)
Age at onset (years)	30.9 ± 9.1
Sex (male/female)	14/32
Years between CSF examination and MRI	10.1 ± 2.0
**Disease type (number of patients)**	
PPMS	5 (11%)
SPMS	10 (22%)
RRMS	31 (67%)
**EDSS**	
At time of CSF examination	2(1.5–2.375)
At time of MRI	2(1–5.875)
**DMT at the time of MRI**	
None	14 (30%)
Interferon-beta	11 (24%)
Fingolimod	4 (9%)
Dimethyl fumarate	4 (9%)
Teriflunomide	4 (9%)
Glatiramer acetate	7 (15%)
Other (alemtuzumab, ocrelizumab, azathioprine)	2 (4%)

Normally distributed data are reported as mean ± SD, non-normally distributed data are reported as median (25–75% interquartile range).

*PPMS* primary-progressive multiple sclerosis, *SPMS* secondary-progressive multiple sclerosis, *RRMS* relapsing–remitting multiple sclerosis, *EDSS* expanded disability status scale, *CSF* cerebrospinal fluid, *DMT* disease modifying therapy.

**Table 2:** Significant associations between OPN measured from CSF and regional brain volumes assessed 10.1 ± 2.0 years later.

Rank (k) n = 40	Segmented structures	p	t	Benjamini–Hochberg critical values (Q = 0.05) Q*(k/n)
1	Right inf-lat ventricle	**0.00005**	5.012	0.001
2	Subcortical gray matter	**0.0006**	−4.03	0.003
3	Left inf-lat ventricle	**0.0013**	3.318	0.004
4	Left ventral diencephalon	**0.0014**	−3.69	0.005
5	Right ventral diencephalon	**0.0025**	−3.425	0.006
6	Right lateral ventricle	**0.0031**	3.345	0.008
7	Supratentorial volume (ventricle-free)	**0.0031**	−3.341	0.009
8	Left lateral ventricle	**0.0036**	3.272	0.010
9	Brain volume (ventricle-free)	**0.0043**	−3.2	0.011
10	Right accumbens	**0.0060**	−3.055	0.013
11	CSF	**0.0060**	3.055	0.014
12	WM lesion volume	**0.0070**	2.991	0.015
13	Cerebral white matter volume	**0.0087**	−2.894	0.016
14	Right cerebral white matter volume	**0.0087**	−2.892	0.018
15	Right cortex volume	**0.0088**	−2.889	0.019
16	Left cerebral white matter volume	**0.0095**	−2.854	0.020
17	Cerebral cortex volume	**0.0115**	−2.768	0.021
18	Right putamen	**0.0126**	−2.727	0.023
19	Left accumbens	**0.0132**	−2.706	0.024
20	Left pallidum	**0.0144**	−2.667	0.025
21	Right thalamus	**0.0147**	−2.658	0.026
22	Left cortex volume	**0.0182**	−2.56	0.028
23	Left putamen	**0.0184**	−2.555	0.029
24	Right hippocampus	**0.0195**	−2.53	0.030
25	Left hippocampus	**0.0258**	−2.4	0.031
26	Right caudate	**0.0267**	−2.383	0.033
27	Left thalamus	0.0465	−2.116	0.034
28	Corpus callosum mid-posterior	0.0505	2.075	0.035
29	Left caudate	0.0609	−1.981	0.036
30	Right amygdala	0.0941	−1.753	0.038
31	Right pallidum	0.1464	−1.508	0.039
32	Left amygdala	0.1818	−1.381	0.040
33	Left cerebellum white matter	0.1947	−1.34	0.041
34	Corpus callosum posterior	0.2913	1.082	0.043
35	Right cerebellum white matter	0.3406	−0.975	0.044
36	Corpus callosum mid-anterior	0.4552	−0.761	0.045
37	Right cerebellum cortex	0.5592	−0.594	0.046
38	Corpus callosum anterior	0.5784	−0.564	0.048
39	Corpus callosum central	0.5877	−0.551	0.049
40	Left cerebellum cortex	0.7219	−0.361	0.050

Table shows the results of multiple linear regression models. The dependent variables were the segmented brain structures and OPN concentration, age, gender and estimated total intracranial volume were used as independent variables. Significant p-values surviving FDR correction are presented in bold.

The study was conducted according to the World Medical Association Declaration of Helsinki and approved by the Regional Ethical Committee of the University of Pecs (7068-PTE 2018). All patients signed written informed consent prior to study procedures.

### Measurement of osteopontin in serum and CSF

After centrifugation, supernatants were stored at −80 °C until further processing. For quantitative detection of OPN concentrations in the serum and CSF samples, a commercially available sandwich enzyme-linked immunosorbent assay (ELISA) kit was used (Human Osteopontin DuoSet ELISA, R&D Systems, Minneapolis, MN). All preparations were performed according to the manufacturer’s instructions. Samples were diluted for analysis (Serum 1:25; CSF 1:100). All samples were run in duplicates. An iEMS MF microphotometer was used for optical density detection at 450 nm (Thermo Labsystem, Beverly MA, USA). The detection limit for the assay was 62.5 pg/mL.

### Magnetic resonance imaging

All subjects were scanned using the same 3 T MRI scanner (MAGNETOM Prisma^Fit^, Siemens AG, Erlangen, Germany) with a standard 20-channel head-neck coil. Brain volumetry was based on a 3D T1 magnetization-prepared rapid acquisition with gradient echo (MPRAGE) sequence acquired according to the Freesurfer’s Morphometry Protocols Guideline (TR/TI/TE = 2530/1100/3.37 ms; Flip Angle = 7°; 176 sagittal slices; slice thickness = 1 mm; FOV = 256 × 256mm2; matrix size = 256 × 256; receiver bandwidth = 200 Hz/pixel).

### Volumetric analysis of the T1-weigted MR images

3D T1 images were fed into volumetric segmentation performed with FreeSurfer v6.0. Details of the procedures are described in previous publications^26,27^. Each dataset was checked within the processing stream to verify the following stages: Talairach transform, skull strip, white matter- and pial surface segmentation, as described in Freesurfer’s Recommended Reconstruction Workflow. The *white matter hypointensities* labels were corrected by hand for all subjects to avoid the mis-segmentation of white matter lesions (T1 black holes) as grey matter. The final volumetric results from Freesurfer were fed into statistical analysis.

### White matter lesion segmentation

LST toolbox version 3.0.0 (Lesion Segmentation Toolbox, https://www.statistical-modelling.de/lst.html) was used to automatically segment cerebral white matter lesions on 3D FLAIR images using the lesion prediction algorithm^28^.

### Statistical analysis

All statistical analyses were performed using SPSS (IBM Corp., Version 25.0. Armonk, NY). For volumetric analysis, multiple linear regression models were employed with the volumes of the segmented brain structures as dependent variable and OPN concentration, age, gender and estimated total intracranial volume as independent variables. The assumptions of multiple linear regression were satisfied, as judged by testing for linearity, independence of errors, outliers, normality assumptions of the residuals, homoscedasticity and multi-collinearity. Significance level was set at p < 0.05. Given the large number of segmented structures in the volumetric analysis, multiple comparisons correction with Benjamini–Hochberg procedure was applied with a conservative q = 5%.

## Limitations

The present study bears limitations inherent to the study design. The main limitation is the lack of initial (baseline) MRI measurements. Without the baseline measurement we cannot state that the association with lower regional brain volumes (and larger ventricles) correspond to brain atrophy per se.
